# School-Children

**Published:** 1862-05

**Authors:** 


### HALL'S JOURNAL OF HEALTH.

**Our Legitimate Scope is almost boundless: for whatever begets pleasurable and harmless feelings, promotes Health; and whatever induces disagreeable sensations, engenders Disease.**

WE AIM TO SHOW HOW DISEASE MAY BE AVOIDED, AND THAT IT IS BEST, WHEN SICKNESS COMES, TO TAKE NO MEDICINE WITHOUT CONSULTING A PHYSICIAN.

**Vol. IX.]** **MAY, 1862.** **[No. 5.**

### SCHOOL-CHILDREN.

MANY an industrious, frugal, hopeful, ambitious, and honorable young man begins life with a wife, and a buoyant determination to build up for himself a character and a fortune. But within ten years he has become a careless, indolent, and shiftless lounger. Physicians have peculiar facilities for observing the inner life and histories of the families in which they practice, and know that the downfall of some of the most promising of them had its beginnings in a sickly wife, the secret of her unhealthfulness not having developed itself until after marriage. In this country, the mass of families set out in life with the calculation to make their own fortunes. Nine tenths of those who are rich to-day, began married life poor, relying on themselves alone, while their coëvals who leaned on "rich relations," or married a wife whose "father" wasn't "in heaven"—hence lived to fail in business, leaving nothing behind him but his debts—have been civilly and socially, if not physically dead many years.

One of the first clouds to obscure the blue sky of many a steady and industrious young man's hopes and ambitions; one of the very first things to open up to his mind the unanticipated fact that marriage is not always the thing it is cracked up to be, is that his wife is an invalid, or is at least often "complaining." If he loves her, this depresses him, makes him uneasy, and he remains at home to watch over her. The next step is to call in a physician; then comes the drug-store; not only involving the actual expenditure of money, but neglect of business. These things are repeated from time to time. Meanwhile the household affairs go behindhand. A servant becomes necessary, or those already in the house very naturally grow more wasteful, indifferent, idle, and impertinent, as soon as they perceive that there is no one to look after them. Home next becomes less inviting than it was. The young mechanic or merchant, wearied in body and mind, does not find that solace and comfort and rest in that home which he very naturally looks forward to, as the night comes on. His wife's want of health distresses him ; the want of tidiness about the house annoys him, while the evident waste is a downright discouragement; and not a great while passes before he settles down in the conviction that it is not worth while for him to attempt to become a rich man. He then begins to feel that the most he can do is to make both ends meet; and the very day a young man gives up all hope of laying up any thing, he is lost, as far as becoming a thrifty citizen is concerned. This is as certain a result, as that beginning to lay up something from his own exertions is the first step toward redeeming any man from thriftlessness and beggary.

But to continue the above history. After a while it is found that not only is nothing accumulated, but there comes the necessity of getting things before the money has been earned to pay for them ; then follows the demon of debt, with its pall of blackness of darkness, its secret eating out all man's sensibilities, its feeling of being beholden to others, its sense of dependence, of inferiority; next, the cringings, the prevarications, the actual falsehoods, self-contempt, recklessness, desperation, liquor, the hospital, death !—leaving behind a poor widow with fatherless children, and destitution staring them all in the face. Next there comes to that widow the fearful, life-long struggle for bread for her little ones! She becomes prematurely old, and her whole existence is one of mental anguish, and bodily privation and toil. There are multitudes of such widows to-day, in all the larger cities of the nation, and not a few in town, village, and country. Mother! look at your sweet daughter of two, or ten, or fifteen years, and think if you would not rather see her dead this very hour, than that she should weave such a history for herself! And now we come to the practical part of it, and urge you to do what you can to lessen the probabilities of such a record as to your darling child, by *educating her to healthfulness!* And you can not begin too soon. Your daughter has entered her sixth or seventh year, or more, and is going to school, leaving home before nine o'clock, not to return until after three. She will necessarily want something to eat about noon. There is nothing better, nothing half so good, as a sandwich—a piece of lean meat of any kind between two pieces of light bread; this is plain and nourishing, is quite enough to break the edge of hunger, without being enough to prevent a good appetite for a regular dinner between three and four o'clock, or later. Such a lunch may be alternated, occasionally, with two or three good, ripe, perfect, juicy apples. If a child is not hungry enough to relish a sandwich or an apple, nature does not really want any thing. If you provide them with what is more inviting, in the shape of nuts, cakes, pies, candies, and the like, they not only are tempted to eat more than is necessary, but are made thirsty. This "fills them up," and by causing more or less a feeling of oppression, indisposes and incapacitates for easy study, while their dinners at home are not so much enjoyed, if indeed the stomach has acquired, since "lunch," vigor enough to perform its duty thoroughly and well; and in proportion as this is not the case, the system becomes dyspeptic, no good blood is made, and the constitution begins to become impaired. If dinner is taken at home after three o'clock, then the child should eat nothing later than dinner, beyond a piece of cold bread and butter, and half a glass of water, or a bowl of milk and bread, or stirabout of Indian or oat-meal, or a piece of bread and butter, with a cup of hot water and milk, sweetened, called "cambrick tea." Such a supper will not be very inviting, and there may be exhibited some feeling of dissatisfaction, but it will have the invaluable effect of allowing them to sleep soundly, and of waking up with a keen appetite for a hearty breakfast, with the after exercise of the day to "grind it up" into perfect, healthful, life-giving blood, to say nothing of the greater ability to study to advantage during the evening and in the early morning. Every mother must have noticed that when a child has a good appetite for breakfast, there is more liveliness, activity, animation, and cheerfulness in going to school; in fact, it is an actual pleasure, when they are to meet kind, conscientious, patient, and considerate teachers—for example, such as Miss Adams, Miss Armitage, and some others in our Twelfth-street school. But suppose you spread a different "supper," and have on the table tea and coffee, and hot rolls or muffins, with nuts and pickles, or chipped beef, preserves and sweet-cakes—they will certainly over-eat, will certainly spend a more or less restless night, will wake up later than usual, more cross, irritable and snappish than is common, will eat little or nothing for breakfast, and will start off to school in a moody, or at least an unlight frame of mind, only to worry the mother and keep her anxious the whole morning, and until the middle of the afternoon. And worse still, poor "hubby" comes in for a share of the unwelcome spoils; because, when from any reason the wife becomes "uncomfortable," she will in too many cases reduce the whole household to a "sameness." Sensible wives are excepted herefrom, being as multitudinous proportionably as—four-leaved clover.

Compel your children to be in bed by the time the clock strikes nine, summer and winter, except on Friday, Saturday, and Sunday nights, and occasionally at other times, in case of parties, weddings, and the like, for amusements and diversions are essential to the well-being of children; besides, it is not well to bring them up to inflexible rules, as if they were machines, or to bring them under the tyranny of Medo-Persian laws, which admit of no repeal or change. This early retiring will do much toward preventing the early ruin of the eyes, by artificial light, and will also secure that liberally abundant sleep and rest to the brain essential to bodily health and mental integrity. It is an undeniable and often-observed fact, that a failure to get abundant sleep lays many a promising child in an early grave, by inflammation of the brain. Too little exercise and too continuous study do the same thing; and when lack of sleep, lack of exercise, lack of out-door air, are combined with insufferable drillings at school, and impossible lessons at home, we can not wonder at the multitude of deaths of children from convulsions, nervous and brain diseases, from debility and wasting away. Let it be remembered that the more sleep a child can get during the night-time, the more quickly and readily and easily will all the studies be comprehended; and to this end, if the child is put to bed at a regular, early hour, the rule should be imperative that it should never be waked up in the morning. Nature will always do that with unerring certainty, just as soon as sufficient sleep has been had to repair the wastes of the preceding day, and provide a stock of strength to be drawn upon for the day to come. This is a beautiful law of our nature, and ought not to be contravened, for it can never be interfered with, with impunity, especially as to the young.

Next to eating and sleeping, the item of most universal application is the proper regulation of the bowels. We will feel a thousand times repaid for writing this article, if every mother who reads it will begin, without a day's delay, to impress upon the mind of every child she has, from four years old and upward, that the omission to go to the privy every day, will never fail to accompany or precede nine tenths of all the sickness of old and young. A good way of drawing a child's attention, and securing its reflection on this subject, is to give it a penny for every day during which it does not fail to go back at least once, explaining at the same time the reasons for it. Another way of deepening the impression is simply to notice the fact yourself, and to call the child's attention to it, how almost invariably when it is too sick to eat, the bowels have missed a day or two; and the other quite as frequent an occurrence, that whatever may be the sickness, (unless in the nature of diarrhea,) when the bowels begin to move, that sickness begins to abate, and the appetite begins to return. By such a course, the child will soon see the connection between health and regularity, and will seldom fail to give proper notice. If a day is about to close and such notice is given, the wise, safe and efficient course is to put the child early to bed without any supper, or if any, a single cup of hot cambrick tea and a piece of dry bread; let the mother sleep with the child, so as to keep its feet warm and the body well covered, which, by promoting perspiration, will aid in unloading the system of the impurities which cause the ailment. Let breakfast be the same as supper, and a dinner at noon of plain gruel or soup, with any kind of cold bread-crust broken into it, at intervals of not less than four hours. Such a course as this will forestall many an attack of sickness, not only as to children, but as to grown persons, because the lighter diet named, gives the system rest; this in turn allows it to gather strength to throw out the commencing accumulations, without the necessity of losing an hour from school or business. It must strike the least thoughtful reader, that if the main outlet for the wastes and surplusage of the system is closed, and yet if the amount eaten is not materially less, there must be a clogging up of the whole machine, and some consequent disaster is as inevitable as the blowing up of a locomotive if steam is kept constantly generating and the escape-valve is firmly closed.

The plan suggested is immeasurably safer and surer than that of administering medicinal remedies to children, however "simple"—that is, familiar—those remedies may seem to be. In four cases out of five, the foundations of life-long ailments, which embitter the whole existence, are laid in habits of constipation originating in the school-room—oftener, perhaps, in a late breakfast, which makes it necessary to hurry off to school, and in the excitement nature's calls are unnoticed, and may remain so until school is called, and time has been allowed for all to settle down quietly at their lessons. But then a recitation is called; then the child "must" wait until the lesson is over; after that the teacher may be too busy; next the child begins to calculate that it will soon be time for dismission. Thus, in one way and another, nature is baffled, but not with impunity; for just precisely in this or similar ways, habits of constipation commence in multitudes, old and young, every day, paving the way for some of the most protracted, some of the most troublesome, some of the most painful ailments known to man. In the earlier stages, and most commonly, there is a complaint of cold feet, of headache, of chilliness, of want of appetite, of no inclination for breakfast of mornings, of dullness, of want of vigor, vivacity, animation; and the child goes off to school, or the adult to business, "more dead than alive," and all creation looks as blue as indigo. The child is fickle, fretful, peevish; the man sullen and *groany;* the woman—my stars! how she does make every body stand about! The servants huddle together in the kitchen, the children will be as mute as mice, and "father," if he has a mite of sense, will quietly edge himself off into all out-doors. If breakfast be taken at seven o'clock, or at seven and a half, during January and February, there need be no hurry for school, and there will be that leisure which allows of deliberate attention to nature's admonitions.

It is well worth while to be at even greater pains to guard our children against falling into habits of constipation, when it is known that such habits lead to dyspepsia, piles, fistula, bilious colic, sick headache, and that "nervousness or neuralgia" which makes life intolerable to so many wives and daughters. Two cases came within our notice in 1861, of an affection, the result of former constipation; for although the bowels had become regular again, the effects remained. To give additional interest to the subject, it may be well to premise, that one was a gentleman of distinction—the president of a college; the other was that of a lady of high culture, of enviable social position, and of great personal attractions. The gentleman seemed worn down by intense suffering. No one would suppose, by the lady's appearance, that any thing was the matter with her; but they do have such a way of hiding things. They may be dead in love with you, for example, and you wouldn't know a word about it! They were both as regular as the morning's coming. But each morning was a martyrdom. The pain attending each evacuation, and continuing for two or three hours afterward, was so intense as to produce utter prostration, and no position was endurable. There was no help for it, but to lie on a bed and agonize for two weary hours, when the sufferings would begin to subside, and finally disappear until the next morning. Such a condition of things as this is fearful to think of. We turned both these cases over to an accomplished surgeon, being out of our line of practice. The gentleman was perfectly cured in a few months; of the lady nothing more was heard.

Every rational woman who has a mother's heart, and who has a daughter at school, will make it a matter of affection and duty to do all that is possible to keep her uniformly and always in a cheerful and hopeful frame of mind, sympathizing with her in her difficulties, and sustaining her in her discouragements. The solicitudes of a child as to a recitation are scarcely less to it than the affairs of an empire to a minister of state. In both cases the whole mind is taken up in the subject. The considerate mother, then, will always send her child to school in the morning with a cheerful, affectionate, and an encouraging good-by; will always give her a kindly greeting on her return, for she may be brought home deformed for life, or dead, or with a long or fatal sickness. Never fail to welcome their return with a glad greeting, with some cheerful remark, or some sympathizing, or loving, or encouraging inquiry; and when they are at home, study to do as little as possible to ruffle their minds in any way whatever, so that they may bring a buoyant helping cheerfulness to their studies. Thus will they literally "run and not weary, and walk and not faint," as to their lessons. Let humane parents bring it home, and see to what little advantage they can study if the mind is ruffled, whether by anxiety or irritation. The aims and ambitions, the rivalries, the difficulties, the mishaps, the failures of the school-room, as completely fill the minds of children, and loom up into the vast, as those of a prime minister; and shame and contempt be upon those parents who, under these circumstances, can be so lost to reflection and the commonest feelings of pity, as to place no restraints upon their ugliness, and harrow up the feelings of their going-to-school children, by an incessant laying down of rules and regulations, by petulant fault-findings on the most trivial occasions, crossing them in matters contemptibly trifling, exhibiting on the most frivolous occasions a petulance and an inconsideration which would disgrace an ignoramus. There is another item of vast, yet of every-day importance, and the necessity of specific attention to it is steadily recurring; it is a thing for which a wise and just parent ought to be on a constant lookout. Do all that is possible to make your child see and feel and believe that its teacher is its friend, and not its enemy. This is especially necessary as to the younger children. Explain to them, by a great variety of illustrations, that the object of the teacher is to benefit them; that it would be a great deal easier to give them little or no attention, to take no notice of their ill-learned lessons, and to let them do pretty much as they liked; but rather than be thus slack, that their teachers take a great deal of trouble to bring them on in their studies, because they know that it will add to the intelligence, respectability, and happiness of the child in after-life; and that although they may not feel it now, they will hereafter, when their teachers are dead and gone, and will often think of their painstaking with affectionate remembrance, and vainly wish that they could be brought back again, that they might show them how thankful they were for their patient and conscientious fidelity, through all their own waywardness and thoughtlessness, in the sunnier days of childhood. There may be times when, from the representations of your children, you may be led to feel that the teacher has acted unjustly or inconsiderately, or even with deliberate partiality or indiscretion. Never countenance representations of this sort to the extent of allowing your child to see that you take sides against its teacher. If you do any thing, make a private application to the teacher, remembering, in the manner of the interview, that that teacher is your equal in position, in intelligence, and in moral worth, and not unlikely your superior when the *results* of a lifetime are summed up; for there is not on this earth a higher responsibility than that of a child's teacher—no greater honor due, than that which belongs to one who performs that duty with patience, ability, and conscientiousness. Special attention is called to this matter, because it is perfectly amazing to note how adroitly a child of even five years of age may "make out a case" against its teacher, by leaving out an item here and there, in the narration. To recapitulate, let any mother who truly loves her daughter, and wishes to educate her for a life of efficiency, happiness, and health, begin at the school-room, and bring up that daughter to regular and temperate modes of eating plain, nourishing food; to regular bodily habits; to early, regular, and abundant sleep; and to cherish for its teachers a steady kindliness, confidence, and respect; all the while religiously and persistently aiming to make home pleasant to your children, so that they may lend their minds to their various studies with greater concentration, cheerfulness, efficiency, and ease.

